# Extraction of Multi-Labelled Movement Information from the Raw HD-sEMG Image with Time-Domain Depth

**DOI:** 10.1038/s41598-019-43676-8

**Published:** 2019-05-10

**Authors:** Alexander E. Olsson, Paulina Sager, Elin Andersson, Anders Björkman, Nebojša Malešević, Christian Antfolk

**Affiliations:** 1 Department of Biomedical Engineering, Faculty of Engineering, Lund University, Lund, Sweden; 2 Department of Hand Surgery, Skåne University Hospital, Malmö, Sweden

**Keywords:** Learning algorithms, Translational research, Biomedical engineering

## Abstract

In contemporary muscle-computer interfaces for upper limb prosthetics there is often a trade-off between control robustness and range of executable movements. As a very low movement error rate is necessary in practical applications, this often results in a quite severe limitation of controllability; a problem growing ever more salient as the mechanical sophistication of multifunctional myoelectric prostheses continues to improve. A possible remedy for this could come from the use of multi-label machine learning methods, where complex movements can be expressed as the superposition of several simpler movements. Here, we investigate this claim by applying a multi-labeled classification scheme in the form of a deep convolutional neural network (CNN) to high density surface electromyography (HD-sEMG) recordings. We use 16 independent labels to model the movements of the hand and forearm state, representing its major degrees of freedom. By training the neural network on 16 × 8 sEMG image sequences 24 samples long with a sampling rate of 2048 Hz to detect these labels, we achieved a mean exact match rate of 78.7% and a mean Hamming loss of 2.9% across 14 healthy test subjects. With this, we demonstrate the feasibility of highly versatile and responsive sEMG control interfaces without loss of accuracy.

## Introduction

The electromyogram (EMG)^1^ is a time signal which describes the bioelectrical activity in skeletal muscles. The morphology of the EMG is associated with the activation, or firing, of motor units during muscle contraction. The signal is acquired by measuring the difference in electrical potential between points in (intramuscular EMG; iEMG) or on the skin covering (surface EMG; sEMG) a muscle or muscle group of interest. A high-density surface EMG (HD-sEMG)^2^ is a form of sEMG where the measurement is typically acquired via a two-dimensional grid of electrodes placed on the skin of the subject. Because sEMG is a non-invasive technique it has since long been successfully applied in clinical routine, most notably for diagnosis of neuromuscular disease^3^. Since EMG is a predictor of muscle forces^4^, an alternative use of sEMG is as a control signal for a system which transforms the myoelectric signal into an executable command for a device, such as a prosthesis^5^, an exoskeleton^6^ or a video game^7^. A system controlled by EMG signals is commonly referred to as a muscle-computer interface (MCI).

For applications of this kind, where the MCI output is to be interpreted as a movement command, it is desirable to have a *natural control scheme*, which means that the sEMG generated by one movement corresponds to MCI output encoding that very movement. To create a device with this property would require a sufficiently accurate computational estimate of the actual mapping between the space of possible sEMG signals and some space of possible movement commands. Such a mapping has proven itself elusive and difficult to model for a variety of reasons. The most important reason is that the neuromuscular processes that occur during muscle activity are inherently very complex and are at best ambiguously described by a sEMG measurement. This complexity is due to the physiological fact that each muscle is composed of a set of motor units, each which in turn is composed of many individual muscle fibers. When activated, each motor unit emits an action potential representing a sum of the electrical fields emanating from all its individual fibers^8^. As such, the signal from each sEMG electrode represents an aggregate of action potentials from adjacent motor units, additionally obfuscated by propagation through muscle-, fat- and skin tissue. Furthermore, the sEMG is notably affected by complicated types of noise, such as motion artefacts and other measurement problems^9^.

Because of the complications described above, most commercially available MCI-reliant products (of which myoelectric prostheses are perhaps the most common) rely on simpler control schemes such as two-electrode proportional control^10^. The raw sEMG has traditionally been assumed to have small discriminatory power in movement classification due to its observed stochasticity, nonlinearity and unpredictability^11–13^. Because of this, previous studies aimed at improving the standards of myoelectric decoding have instead often relied on manual feature engineering^14^, where each sEMG channel, commonly segmented into time blocks^15^, is condensed into a set of more robust and descriptive numeric values called *features*. Such features can subsequently be used to train and evaluate a classifier. However, the creation of discriminatory sEMG features is, as in most applications of inferential statistics, a labor-intensive process that requires from the designer a good understanding of the physics of the specific problem domain. More recently, Geng *et al*.^16^ defined the concept of a *sEMG image* as a grayscale image with intensity values proportional to the raw HD-sEMG measured at a single time instant and achieved unprecedented performance by applying a deep learning image classification algorithm directly on such data. From such results it can be postulated that spatial patterns correlated with movement information exists in the instantaneous raw HD-sEMG and allows for exploitation by a classifier.

Independent of feature extraction, modeling the relationship between myoelectric activity and movements is often, quite naturally, framed as a multi-class statistical classification problem. In the relevant case of hand movement recognition, the class set would consist of the set of detectable movements, while the observation instances are represented either by raw sEMG or sEMG features. While certainly a useful framework, a problem inherent to multi-class classification approaches is that the performance of any multi-class classifier devised for movement recognition by necessity decreases as the number of classes increase. This is mainly due to the fact that the EMGs associated with similar (in the sense of recruiting mutual motor units) movements are highly correlated^17,18^ and thus even a sophisticated classifier used in conjunction with well-crafted features eventually lack sufficient discriminatory power. In this sense, the cardinality of the detectable movement s*et al*ways represents a compromise between classification quality (i.e. control robustness) and versatility (i.e. range of detectable movements).

In this paper we propose an alternative approach, designed to mitigate the issue of interclass correlations. We model a hand gesture not as a monolithic class, but as a combination of elements from a given set of simpler ‘basis’ movements. In the language of multi-label machine learning^19^, the necessary and sufficient condition for a given hand movement is constituted by the presences and absences of certain mutually independent *labels*. The set of detectable movements is thus the set of possible movement label combinations. The potential value provided by the proposed framework is thought to lie mainly in its potential for scalability and stability. Importantly for prosthesis control applications, the detection of each label can be viewed as a separate classification task. Thus, in contrast to the traditional multi-class (single-label) approach, the introduction of additional classes (labels) does not directly compete for performance with those already existing. In a related sense, the multi-label approach presents a new source of stability, namely that of partial errors. When a single-label classifier infers movement intent erroneously, the prediction is by definition wholly unwanted and can result in, for example, erratic prosthesis behaviour. The output of a multi-label classifier, on the other hand, might provide a largely stable experience for the user if the majority of labels are correctly predicted most of the time, even if some individual labels are sometimes mispredicted. Lastly, and perhaps most notably, a multi-label model of this kind might ideally be able to learn to infer compound movements consisting of labels combination that do not explicitly occur in its training data, thereby massively inflating the effective range of performable movements. This ability would however require the modulation of the sEMG associated with one label combination induced by other, not previously observed, label combinations to be negligible; a property not explicitly investigated in this study.

**Figure 1:**
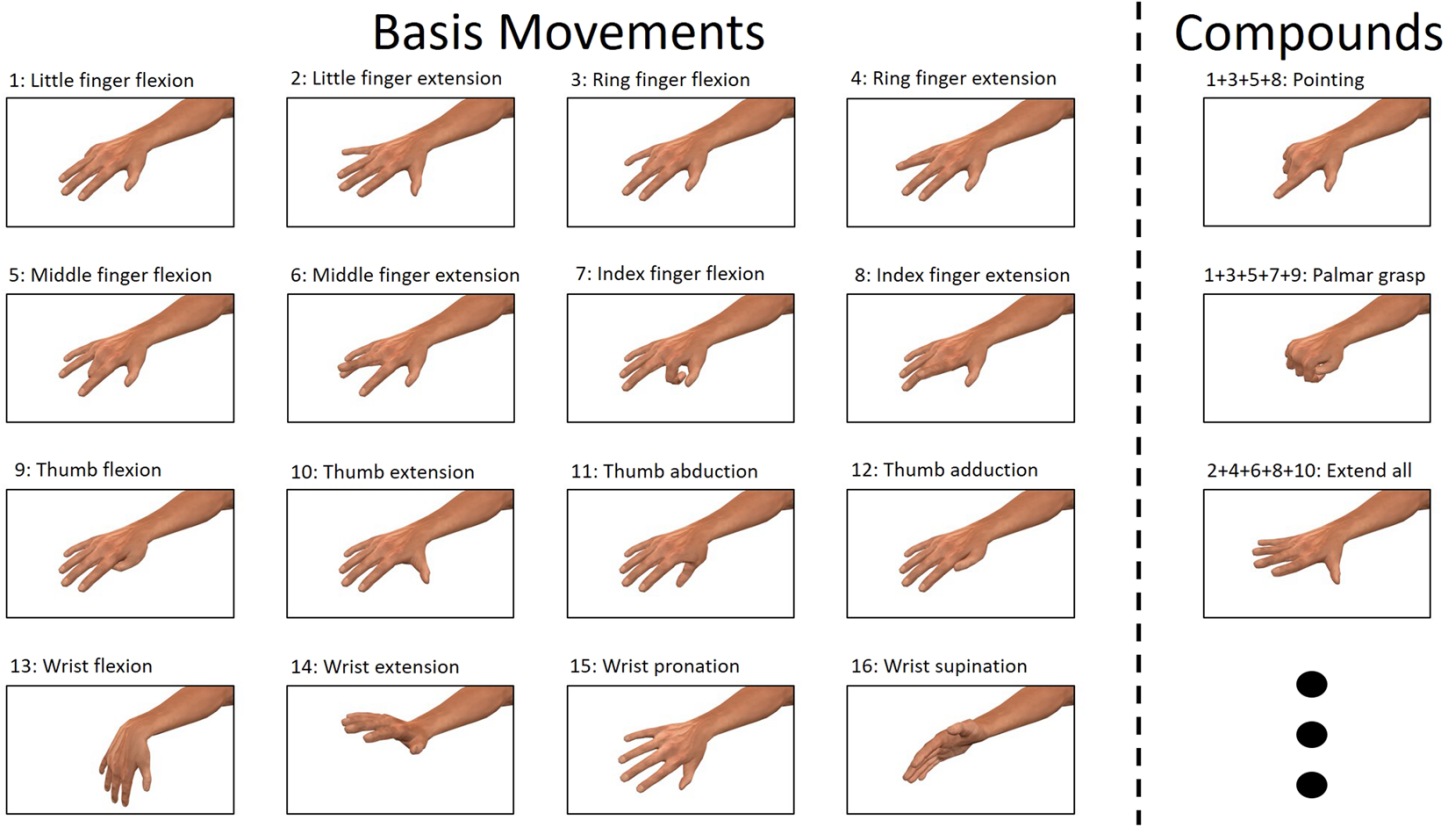
(left) Visualization of the label basis, constituted by 16 movements, used for multi-label classification and (right) some examples of compound movements constructed by combining labels.

For our experiments we adopted the use of 16 labels, shown in Fig. 1, representing flexion and extension of all digits and the wrist, thumb abduction and adduction and wrist pronation and supination. These labels were selected on the assumptions that (1) they represent movements that utilize different forearm muscles or muscle compartments^20^ and (2) they, when allowed to superpose, adequately capture the major degrees of freedom possessed by the human hand. By virtue of the first assumption, they should each generate HD-sEMG signals that contain discernible patterns that, when compared pairwise across labels, are distinct enough to allow for recognition of individual labels. We implemented a deep learning^21^ classifier in the form of a convolutional neural network (CNN)^22,23^ with the purpose of detecting these movement labels in the HD-sEMG signal. The detection of more complicated movement is in our framework equivalent to simultaneously detecting multiple movement labels separately; some examples of such ‘compound’ movements that incorporate multiple active labels are shown in Fig. 1. To allow for exploitation of both spatial and temporal signal patterns, the classification procedure is performed on rescaled images with time-domain depth, i.e. sequences of consecutive sEMG images; analogous to short clips of sEMG ‘video’. Previous related work^24^ has been successful in demonstrating the efficacy of methods where the time-varying spatial distribution of acquired HD-sEMG is used in order to efficiently generate volitional movement commands. However, the use of structured 3-dimensional input volumes composed of stacked time slices; each depicting the instantaneous muscle state and together leveraged for the purposes of decoding movement intent, has no precedent in the MCI literature. While deep neural networks, particularly those of the convolutional kind, have been successfully utilized for classification of sEMG in the past^16,25–28^, to the best of our knowledge no work has been produced to date where EMG movement decoding is treated as a multi-label classification problem.

Hand prostheses that allows for multiple degrees of freedom have existed for some time (see, for example, the Bebionic hand, Michelangelo from Ottobock and the LUKE arm from Mobius bionics). However, such prostheses are typically interacted with via sequential control strategies reliant on predetermined remnant muscles contraction patterns and as such do not operate with a natural control scheme. Development of natural control algorithms capable of naturally utilizing the large number of available degrees of freedom is therefore positioned well to be absorbed by the growing number of mechanically very sophisticated upper limb prostheses.

## Methods

### Data acquisition

**Figure 2:**
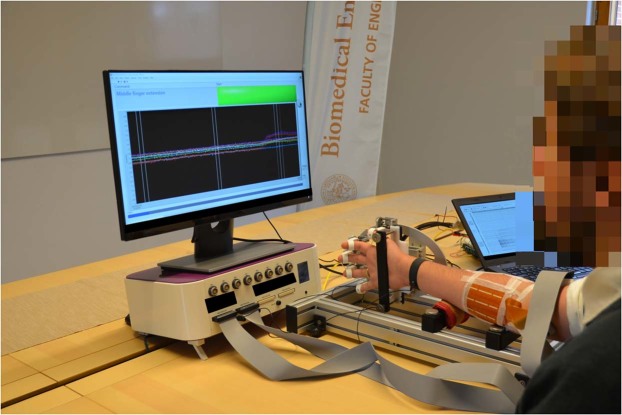
The experimental setup of the acquisition system.

14 adult and able-bodied subjects (9 male and 5 female, age range 25–57 years, median age 37 years) participated in this study. The study was approved by the Regional Ethical Review Board in Lund, Sweden and was conducted according to the tenets of the Declaration of Helsinki. All participants gave their informed written consent. During acquisition, two 8-by-8 electrode arrays with a 10 mm inter-electrode distance (ELSCH064NM3 from OT Bioelettronica, Turin, Italy) coated with conductive gel were attached to the volar part of the right forearm of the subjects. The two electrode arrays covered the skin over the extensor digitorum communis (EDC) and flexor digitorum profundus (FDP) muscles, respectively. The right hand of the subject was subsequently placed inside a custom-built rig where the forearm was comfortably resting, and the hand fixed to the rig while allowing for isometric contractions of the muscles corresponding to the set of movement labels defined for the recording protocol. While seated comfortably in a chair the subject was instructed, through a graphical user interface on a computer screen, to perform a sequence of hand movements as is shown in Fig. 2. Each individual movement lasted for 5 seconds and was repeated 5 times, with 5 seconds of rest in between each movement repetition. The onset of each new movement was accompanied by a sound cue.

Because the number of possible movement label combinations was far too large (2^16^ = 65536) to be exhaustively explored in a reasonable timeframe, a subset of movement combinations was selected on the criterion that the movements should be representative of the most commonly performed hand gestures in a realistic environment^29,30^. Beyond the single label movements, every 2-label combination was recorded, excluding movements containing finger extension together with finger flexion, or with two digits separated by one or more intermediate digits. In addition, some commonly used 3, 4 and 5 label combinations were manually selected to be included: Extension of all finger, flexion of all fingers (Palmar grasp), flexion of digits 2 to 5 (i.e. excluding the thumb), Palmar grasp + pronation of wrist, extension of index finger + flexion of digits 3–5 (pointing), flexion of thumb + flexion of index finger + flexion of middle finger (3-digit pinch), 3-digit pinch + pronation of wrist, flexion of thumb + flexion of index finger (key grasp) and key grasp + pronation of wrist. This constituted 65 distinct movements in total, representing a total recording session time of approximately 1 h for each test subject.

The sEMG was amplified and sampled with an OT Bioelettronica Quattrocento (OT Bioelettronica, Turin, Italy) with a bipolar measurement scheme and a sampling frequency of F_s_ = 2048 Hz. Prior to sampling, a 10–900 Hz analogue bandpass filter was applied to each channel. A LabVIEW (National Instruments, Austin, TX) application was implemented to synchronously acquire the sEMG and the concurrent movement stimulus label set at each time sample.

### Preprocessing

Once acquired, the HD-sEMG time series were digitally filtered channel-wise, first with a 2:nd order Butterworth band stop filter (48–52 Hz) for removal of power line interference and thereafter with a 20:th order Butterworth band pass filter (20–380 Hz) for suppression of noise. Samples coinciding with moments of rest were discarded at this point, justified by the assumption that rest state detection is computationally simple^31^ and thus might undeservedly improve the performance of our classification scheme. In addition, samples corresponding to the first and last second of each 5 s movement repetition were discarded to eliminate effects from transient signal behavior.

The 128 filtered signals were for each sampled time point restructured into matrices of shape 16 × 8 with element positions corresponding to relative electrode placement. These matrices, each representing a single time instant, were individually linearly rescaled into the range [0, 1], where 0 and 1 represent the smallest and largest measured voltage, respectively, across the electrode array at the time of sampling. The resulting normalized matrices could subsequently be interpreted as digital grayscale images. With this method, a specific pixel (i.e. matrix element) value in the resulting images does not necessarily correspond to the same measured voltage across all images. The purpose of this preprocessing procedure was to extract only the spatially structured pattern of motor unit action potentials across the muscles of interest; information which was conjectured to be of greater discriminatory utility than raw myoelectric voltages. Compared to channel-wise or otherwise inter-sample normalization methods, the per-image approach taken here has the additional benefit of temporally isolating the detrimental impact of high absolute value outlier samples (e.g. noise spikes). The images were grouped into sequences of consecutive sampled time points via a sliding window of 24 samples in length and with 12 samples (50%) overlap. Thus, each sequence represents a time length of 23/*F*_*s*_ ≈ 11 ms. The window size and overlap preprocessing hyperparameters were selected ad-hoc, justified by the fact that they represent a minimal decision delay, operate at the (presumably) relevant timescale of sEMG fluctuations and generate a sufficiently large set of image sequence instances (approximately 110000 instances per test subject) for later training and testing of the classifier. In the last preprocessing step, each image sequence was assigned a ground truth label set of 16 bits, where each bit encodes the presence or absence of a certain label during that time interval, determined by a label-wise majority vote over the 24 sampled label sets of the sequence. Majority voting is not strictly necessary within the presented experimental framework, as we have discarded all transient signal sections with ambiguous movement affiliation, but rather serves as a universally applicable method for compressing the sequence of label sets of the window into a single label set.

The resulting image sequence instances were distributed into a training, testing and validation set as follows: instances from the 2:nd and 3:rd repetitions of each movements were used for training, instances from the 4:th repetition for testing and instances from the 5:th and final repetition for validation. Image sequences originating from the 1:st repetition of each movement were discarded since they for some subjects were wrongly labeled because the subjects occasionally forgot to perform the new movement and instead continued with the preceding movement. In all cases when a mistake occurred, an experiment supervisor successfully spotted the error and notified the subject before the 2:nd repetition began, thus preserving the integrity of repetition 2–5 for all movements and subjects. With the outlined procedure, the training, testing, and validation sets are all balanced w.r.t. the number of unique movement combinations (but not necessarily the number of individual basis movement labels). The preprocessing described here was performed via the use of custom MATLAB (The MathWorks Inc., Natick, MA) scripts.

### CNN model

**Figure 3:**
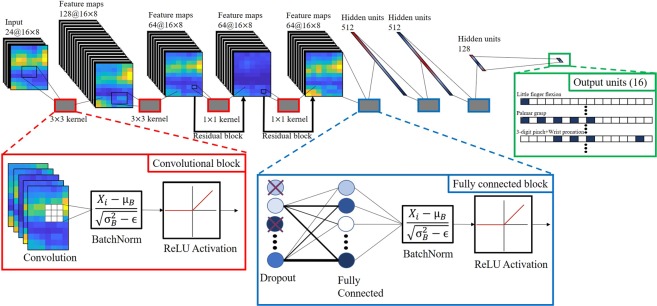
Illustration of the topology of the deep convolutional neural network.

The structure of the CNN used in the current study was inspired by the one used by Du *et al*.^25^ and is illustrated in Fig. 3. The topology and hyperparameters of the network described below were found empirically via evaluation on previously collected data and were not subject to change at any point during the current study. The input layer is a tensor of size 16 × 8 × 24, representing one HD-sEMG image sequence generated in accordance with the preprocessing steps described above. It is followed by 4 convolutional blocks, connected in a feed-forward configuration, with 128, 64, 64 and 64 filters with kernel sizes 3 × 3, 3 × 3, 1 × 1 and 1 × 1, respectively. Each convolutional block follows a Convolution-BatchNorm^32^-rectified linear unit (ReLU)^23^ structure, and the 3:rd and 4:th convolutional blocks have residual blocks^33^ incorporated to facilitate convergence. The output from the last convolutional block is fed through a cascade of 3 fully connected blocks with a dropout^34^-fully connected (FC)-BatchNorm-ReLU structure with 512, 512 and 128 output neurons, respectively. All dropout layers have the dropout probability hyperparameter set to 0.5 during training. The last FC layer contains 16 output neurons, one per label, and a final sigmoid activation layer for generating label probabilities. To generate categorical label predictions, the 16 outputs of the final layer is simply compared to a probability threshold value *t* ∈ [0, 1]. If the i:th output element is greater than *t*, then the i:th label is predicted as present, otherwise absent. A higher threshold intuitively represents a higher requirement of prediction certainty on the part of the network to include a label in a prediction. The selection of *t* presents a directly impactful avenue of model tuning that is unavailable to (single-label) multi-class methods. In general, the threshold could be set separately for each label to achieve an arbitrarily low false positive- *or* false negative rate, but likely at the cost of a corresponding increase of the other. As such, the selection of threshold should reflect the evaluated importance of type I errors (i.e. ground truth absent labels predicted as present) relative to the importance type II errors (i.e. ground truth present labels predicted as absent). For example, in prosthesis control applications an argument could be made for a greater imperative to minimize the former (false positives), as such errors are more likely than the latter (false negatives) to be perceived by a user as directly antithetical to control stability. In our experiments we sidestep these considerations for the sake of brevity by adopting the same threshold across all labels; namely that which generates the highest exact match rate on the validation set (as is determined during model fitting). The neural model was implemented in Python 3.5 with the use of TensorFlow^35^, an open-source machine learning library capable of running on graphics hardware. The model contains 4636560 learnable parameters.

### Model fitting

For backpropagation^21,36^, two different loss functions were evaluated: binary (per-label) cross-entropy loss^37^ and BP-MLL loss; a loss function developed by Zhang *et al*.^38^ specifically for training neural networks with multi-labelled output. Both loss functions were used in conjunction with a weight decay of 2∙10^−6^. During training, the Adam algorithm^39^ with a learning rate of 0.03, mini-batch size of 3000, β_1_ = 0.9, β_2_ = 0.999 and ε = 10^−8^ was used for optimization. All learnable parameters of the network were initialized via random sampling from a truncated normal distribution with zero mean and unit variance. Training proceeded for up to 250 epochs; after every even-numbered training epoch the model was evaluated on the validation set. If no improvement over the best validation exact match rate so far was detected for 5 consecutive validations the training was halted prematurely to avoid overfitting (so-called *early stopping*). The order of the examples in the training set was randomly reshuffled prior to the onset of each new epoch. The classification probability threshold of the final network layer is determined by evaluating the fitted model on the validation set and selecting the threshold value, out of 11 candidate values sampled uniformly between 0 and 1, that maximizes exact match rate. Training was performed once per test subject training set with the fitted model applied once on the test set of the same subject to obtain performance metrics. The optimization procedure lasted for approximately 1–2 h per test subject on a desktop computer equipped with a GeForce GTX 1070 GPU (NVIDIA, Santa Clara, CA). The average time required for extraction of labels from an image sequence instance (i.e. a single network forward-pass) was 1.6 ms.

### Comparison with a single-label classification scheme

In order to verify the viability of our method compared to a more conventional single-labeled classifier operating under similar conditions, an alternative network topology was evaluated on the same data set. The last two layers of this network were set as a FC layer with 65 output units (representing the set of unique recorded compound movements) followed by a softmax activation layer. Categorical prediction was performed by finding the output unit with the largest activation, as is convention in (single-label) multi-class classification, and thus no thresholding was neither required nor possible. During training, categorical cross-entropy^37^ was used as the loss function to be minimized. With these final layers and loss function except, the network and the optimization procedure were identical in structure and hyperparameter selection to the model presented above. The BP-MLL loss, due to being inherently multi-label, was not utilized to train this network. As was the case for the multi-label network, training and evaluation was done once per test subject.

## Results

A set of performance metrics relevant to multi-label classifications were selected to benchmark the predictive power of the fitted model. These were calculated once per subject on the entirety of their respective test set; the values reported in the following sections were acquired by computing the arithmetic mean and standard deviation for each metric over all subjects. Compiled results are presented in Table 1, and all results presented per subject, label and compound movement are available in Supplementary Tables 1–3. For all subjects, the optimal classification probability threshold of the neural model (derived via iterated evaluation on the validation set as described in the previous section) was determined to be 0.5 and 0.9 when trained with cross-entropy loss and BP-MLL loss, respectively. The metrics reported here were generated by models operating at these thresholds.

**Table 1:** Average performance metrics of the classification process across subjects.

	EMR	HL	JI	P	R
Cross-Entropy Loss	0.787 ± 0.064	0.029 ± 0.011	0.847 ± 0.055	0.894 ± 0.040	0.878 ± 0.054
BP-MLL Loss	0.695 ± 0.086	0.034 ± 0.013	0.827 ± 0.055	0.856 ± 0.049	0.891 ± 0.044

The range of each value represents its standard deviation across all subjects.

### Exact match rate

The Exact Match Rate (EMR)^40^ represents the proportion of observed image sequence instances where every single label is correctly predicted by the classifier:

1 $$EMR=\frac{1}{N}\sum _{t=1}^{N}1({p}_{t,i}={y}_{t,i}\forall i);\,\,{\rm{EMR}}\in [0,1]$$

where N denotes the training set cardinality, and *p*_*t*,*i*_ and *y*_*t*,*i*_ denotes the prediction and ground truth, respectively, of the i:th label in the t:th test instance (1 if label is present, 0 if not). 1(∙) is the indicator function, returning 1 in the case that its argument is a true condition, 0 otherwise. While closely related to the accuracy metric of conventional single-label classifiers, EMR is in a general sense much stricter the larger the set of possible labels is; a single mispredicted label in one instance marks it as failed when computing the EMR.

In the comparative case of a classifier randomly predicting each label as either present or absent with uniform probability, the expect an EMR baseline of ${2}^{-{\rm{Q}}}={2}^{-{\rm{16}}}=\frac{1}{{\rm{65536}}}\approx {\rm{0.000015}}$. We achieved a mean EMR of 0.788, standard deviation σ = 0.079 when the network was trained with cross-entropy loss. With BP-MLL loss training, the resulting numbers were reduced to 0.694, standard deviation σ = 0.084. With cross-entropy loss, individual subjects reached EMR values as high as 0.879 and as low as 0.607.

### Hamming loss

The Hamming Loss (HL)^40^ operates on each label independently by measuring the ratio of wrongly predicted individual labels to total number of labels over all observed instances:

2 $$HL=\frac{1}{N}\sum _{t=1}^{N}\frac{1}{q}\sum _{i=1}^{Q}1({p}_{t,i}\ne {y}_{t,i})=\frac{fp+fn}{tp+tn+fp+fn};\,{\rm{HL}}\in [0,1]$$

where Q = 16 is the number of possible labels. *tp*, *tn*, *fp* and *fn* denote true positive labels, true negative labels, false positive labels and false negative labels, respectively. In contrast to the other metrics presented here, a lower HL corresponds to more correctly predicted labels and is thus desirable. By its definition, HL can never exceed 1-EMR, but might be considerably smaller if the classifier often partially misclassifies instances.

In the comparative case of a classifier randomly predicting each label as either present or absent with equal probability, the expected HL would reach a baseline of 0.5. We achieved a mean HL of 0.031, standard deviation σ = 0.012 when the network was trained with cross-entropy loss. With BP-MLL loss training, the resulting numbers increased to 0.034, standard deviation σ = 0.012. With cross-entropy loss, individual subjects reached HL values as low as 0.017 and as high as 0.057.

### Jaccard index

The Jaccard Index (JI)^41^, sometimes referred to as *intersection-over-union*, is a statistic for measuring similarity between two sets; A and B:

3 $${\rm{JI}}({\rm{A}},{\rm{B}})=\frac{|{\rm{A}}\cap {\rm{B}}|}{|{\rm{A}}\cup {\rm{B}}|}$$

here we calculate its value between the set of predictions from the test set and the test set ground truth by considering each label for each instance as a possible set element:

4 $${\rm{JI}}=\frac{{\sum }_{t=1}^{N}{\sum }_{i=1}^{Q}1({y}_{t,i}=1\wedge {p}_{t,i}=1)}{{\sum }_{t=1}^{N}{\sum }_{i=1}^{Q}1({y}_{t,i}=1\vee {p}_{t,i}=1)}=\frac{tp}{tp+fp+fn};\,{\rm{JI}}\in [0,1]$$

JI is presented as it represents a simultaneously global and fine-grained measure of classifier performance. In our experiments, we achieved a mean JI of 0.840, standard deviation σ = 0.056 when the network was trained with cross-entropy loss. With BP-MLL loss, the resulting numbers were reduced to 0.827, standard deviation σ = 0.053. With cross-entropy loss, individual subjects reached JI values as high as 0.908 and as low as 0.708.

### Precision and recall

To investigate the possibility of classifier biases, brought about by an unbalanced training set, we calculate the Precision (P) and Recall (R) metrics^19^ commonly used in binary information retrieval tasks^42,43^. Just as for the HL and JI, we extend the definition of these metrics to the multi-label problem domain by viewing each classification as Q = 16 independent binary instances:

5 $$\begin{array}{c}P=\frac{{\sum }_{t=1}^{N}{\sum }_{i=1}^{Q}1({y}_{t,i}=1\wedge {p}_{t,i}=1)}{{\sum }_{t=1}^{N}{\sum }_{i=1}^{Q}1({p}_{t,i}=1)}=\frac{tp}{tp+fp},R=\frac{{\sum }_{t=1}^{N}{\sum }_{i=1}^{Q}1({y}_{t,i}=1\wedge {p}_{t,i}=1)}{{\sum }_{t=1}^{N}{\sum }_{i=1}^{Q}1({y}_{t,i}=1)}\\ \,=\,\frac{tp}{tp+fn};\,{\rm{P}},{\rm{R}}\in [0,1]\end{array}$$

**Figure 4:**
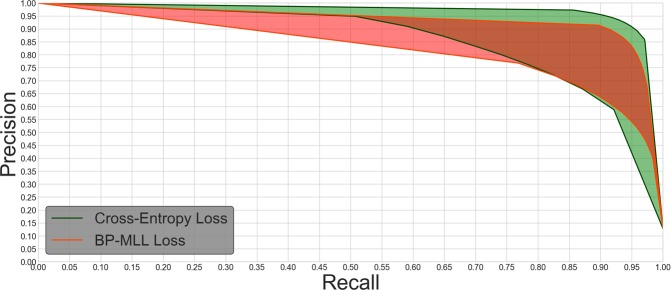
Precision-recall curves. The colored regions represent the different loss functions, with upper and lower bounding curves of each region corresponding to the subjects with highest and lowest EMR, respectively. The curves were plotted parametrically by linearly interpolating precision and recall calculated at 11 equidistantly spaced label detection probability thresholds between 0 and 1.

Precision represents the fraction of all predicted labels that are truly present, while recall represents the fraction of all truly present labels that is predicted as present. Over some family of classifiers with similar HL, it is therefore usual to observe an inverse relationship between P and R: A more ‘lenient’ classification scheme will likely retrieve more of both correct and incorrect labels, while a ‘strict’ scheme will retrieve fewer of both^44^. A high value of both P and R indicate that the classifier indeed exerts discriminatory power and does not simply capitalize on unbalanced data w.r.t. label abundance. The relationship between precision and recall for varying label probability thresholds in the approach taken here is presented in Fig. 4.

In our experiments, we achieved a mean precision score of 0.890, standard deviation σ = 0.042 and mean recall score of 0.868, standard deviation σ = 0.055 when the network was trained with cross-entropy loss. With BP-MLL loss, the resulting numbers were instead P = 0.852 (σ = 0.049) and R = 0.891 (σ = 0.041).

### The single-labeled network

When the modified network was used to predict a single class out of the 65 recorded compound movements, only the accuracy was calculated for comparison as our selected performance metrics have no clear counterpart for single-label classification performance. Over all subject, the mean accuracy achieved was measured as 0.7813, standard deviation σ = 0.068. Individual subjects reached accuracies as high as 0.855 and as low as 0.620.

## Discussion

The main aim of this study was to assess if it is possible to extract information from HD-sEMG measurements that allow for decoding of several independent hand and wrist movements simultaneously. To investigate the feasibility of such a multi-label classification approach, we implemented a deep convolutional neural network to detect up to 16 possible movement labels when given a 24 samples long sequence of sEMG images with a sampling rate of 2048 Hz. With all resulting metrics vastly outperforming a random guessing baseline, the method used in the current study can reliably be said to succeed in its task of extracting information from movement-specific spatiotemporal patterns present in the HD-sEMG. Furthermore, precision and recall scores indicate that this result is not an effect of bias induced by label imbalance or scarcity. Across all performance metrics cross-entropy loss proved slightly to moderately superior to BP-MLL loss for model convergence, despite the latter being specifically developed for use in multi-label models. This could possibly be an effect of plateaus on the high-dimensional function surface as has been previously pointed out^45^ as a possible problem with BP-MLL loss.

In general, comparing the results from the current study with prior studies concerned with sEMG decoding is not straightforward, mainly since previous work has exclusively operated within a single-label framework, i.e. with a smaller set of unique classes and thus with a higher expected performance baseline. Momentarily ignoring the issues presented by comparing single-label and multi-label classifiers, a fair comparison of performance can still only ever be made between classifiers with a similar number of inferable classes. Even so, when trained on data from our experiments and tested on the same subject, EMR values close to, and sometimes higher than, accuracies of single-label approaches were produced^26,46,47^, despite our method being able to represent many more movements (65 unique movements as demonstrated in this study, and possibly many more of the untested compound movements) and EMR being a much stricter measure of performance than accuracy. A comparison with our own single-label classifier verifies that the multi-label approach does not carry with it any discernible reductions in performance to negatively offset the benefits argued for in this paper (scalability, stability and tunability), and additionally validates our method of utilizing raw sEMG image sequences for predictive purposes. Our results thus indicate that encoding hand movements with a multi-label framework could be a useful abstraction for modeling the complex relationship between the spatial and temporal variations of the sEMG and gestures constituted by multiple degrees of movement freedom.

Despite these encouraging results, some limitations on the part of our methodology need to be addressed before methods such as the one under investigation here can be implemented for use in practical circumstances, clinical or otherwise. Due to the scope of this study, data from each subject was collected during a single recording session. The presented results as they stand thus do not guarantee robustness against long-term signal variations, e.g. slight translation of electrode position over time. Further studies in this area would need to be designed to quantify and counteract such effects, or otherwise be able to tolerate small differences between the distributions generating the training-time data and the inference-time data.

The total recording session time of 1 h followed by the 1–2 h of network training necessary to fit the model might be seen as prohibitively long for the method to be of realistic utility. However, only 2 out of the 5 recorded movement repetitions were used for the subsequent fitting of the model. Hence, even within the current framework, a much more manageable recording time; less than half of that in our experiments, would suffice in order to create classifiers on par with those presented in this paper. Notably, the severity of these time constraints depends heavily on the issue of stability over time discussed in the previous paragraph, as the rate of classifier performance deterioration will determine the required frequency of recalibration. If recalibration is required often, the time required for each recalibration session must be short if the method is to have any practical feasibility. Conversely, if recalibration is only rarely required, the recording training procedure could be allowed to last for much longer. This puts further emphasis on the importance of stability over time as an object of inquiry in future studies.

One additional area of concern is that of the requirement of our method on inference time memory and computational complexity. While the extraction of the label set from an image sequence is performed in a shorter time (<2 ms) than the time between consecutively acquired image sequences (~6 ms), and much shorter still than would be required in a real application (~100 ms)^15^, our setup has access to computational resources considerably larger than what can always be expected to be available. Future studies should focus on finding more computationally efficient multi-label classifier architectures to allow for utilization in important but resource-limited applications such as myoelectric prostheses and other types of wearable technology.

In the approach taken here, the selection of labels was based on the empirical assumption that individual finger and wrist movements should be statistically separable while also providing a good approximation of the movement of the hand and wrist state. In the future, it could be of interest to investigate more systematic approaches for the delimitation of the degrees of freedom of the hand, perhaps via the application of unsupervised machine learning, e.g. sparse autoencoders or self-organizing maps. It is likely that better basis movement labels, in the sense of generating more distinct and separable sEMG patterns while their combination space still adequately span the space of performable hand and wrist movements, can be designed.

One intriguing possibility of multi-label classifiers that was not explicitly investigated in this study is that of generalizability to unobserved label combination. Further studies should determine how well a classifier could be made to generalize learned patterns for this task. To be successful with such an approach would however require some way of ensuring that the (likely highly nonlinear) modulation of the sEMG caused by the introduction of unseen label combinations is sufficiently small to not disruptively violate the learned classification boundaries.

In closing, the topology, hyperparameters and optimization procedure of the network itself, while clearly functionally sufficient for achieving the goals stated here, can doubtlessly be improved upon in future work. As successful CNN design heuristics are lacking in the area of myoelectric pattern recognition, it could be of interest to investigate more technically sound methods of topology selection, e.g. supervised optimization procedures such as genetic algorithms.

## Supplementary information

S1

S2

S3

## Data Availability

Data collected during and code written for this study is available from the corresponding author on reasonable request.
